# Mothers’ Experiences and Perceptions of Facility-based Delivery Care in Rural Ethiopia

**DOI:** 10.1177/11786329211017684

**Published:** 2021-05-18

**Authors:** Elin Mordal, Ingrid Hanssen, Andargachew Kassa, Solfrid Vatne

**Affiliations:** 1 Molde University College, Specialized University in Logistics, Molde, Norway; 2 Lovisenberg Diaconal University College, Oslo, Norway; 3 Hawassa University, Hawassa, Ethiopia

**Keywords:** Women, childbirth, primary healthcare, person-centered care, rural, Ethiopia, interviews, observation

## Abstract

In Ethiopia, delivery wards are a part of primary healthcare services. However, although the maternal mortality rate is very high, approximately 50% of mothers use skilled birth attendants. This study focused on how women in a rural southern district of Ethiopia experience maternity care offered at the local delivery wards. In this qualitative, exploratory study, 19 women who had given birth in a healthcare facility were interviewed in 2019. Individual in-depth interviews were supplemented with observations conducted at 2 different delivery wards in the same district in 2020. Two main themes emerged from the thematic content analysis: increased awareness and safety were the primary reasons for giving birth at a healthcare facility, and traditions and norms affected women’s birth experiences in public maternity wards. The main shortcomings were a shortage of medicine, ambulance not arriving in time, and lack of care at night. For some women, being assisted by a male midwife could be challenging, and the inability to afford necessary medicine made adequate treatment inaccessible. Providing continuous information gave the women a certain feeling of control. Strong family involvement indicated that collectivistic expectations were key to rural delivery wards. The healthcare system must be structured to meet women’s needs. Moreover, managers and midwives should ensure that birthing women receive high-quality, safe, timely, and respectful care.

## Background

Primary healthcare facilities are important elements of the current Ethiopian maternity care system, which has undergone significant structural changes in recent years.^1^ Before the worldwide focus on reproductive health and maternal mortality, home births were common in rural areas of Ethiopia, with support from traditional birth attendants lacking formal healthcare training and female relatives or neighbors.^2^ Strengthening the primary healthcare system is expected to further reduce the maternal mortality rate and improve maternal health outcomes.^3^

Maternity care in Ethiopia is structured into a 3-tier system where the levels are interconnected through a referral system and based on a central-peripheral management model. At the local level in rural areas, birthing women are cared for by primary hospitals or healthcare centers. In Ethiopia, attention to antenatal care (ANC) is increasing. The aim is to improve pregnant women’s health and to teach them about the benefits of facility-based childbirth. Health extension workers (HEWs) are prepared to strengthen the utilization of facility-based childbirth. These are local women who prepare groups of pregnant women and are trained to conduct preventive healthcare and individual follow-up before childbirth in the local community as a part of ANC. The HEWs also inform pregnant women about the benefits of giving birth in a maternity ward and thus contribute to the implementation of the authorities’ goal that all women should give birth in healthcare facilities and not at home anymore.^4^

A systematic review from Ethiopia has revealed that women who attend ANC are more likely to give birth in healthcare facilities and attend postnatal care.^5^ Although many pregnant women receive ANC and are informed about the safety of giving birth assisted by skilled birth attendants, many opt for home delivery.^6^ However, the quality of professional ANC services seems to promote institutional delivery.^7^

The Ethiopian National Reproductive Health Strategy describes compassion, respect, care, empathy, and trust, together with the creation of an environment for informed decision-making as crucial for an acceptable quality of service.^8^ This presupposes patient-centered care (PCC), where patients are involved in a way that promotes informed choices and shared decision-making. The concept of PCC was originally described by Edith Balint in 1969 as “understanding the patient as a unique human being” but the concept has evolved since and has gained a central place in all aspects of professional nursing.^9^ Santana et al. call for a more holistic approach to care, incorporating various dimensions of well-being, including patient context, individual expressions, preferences, and beliefs. They developed a conceptual framework of PCC, based on the Donabedian model, which classifies healthcare improvement into 3 categories: structure, process, and outcome.^9,10^

*Structure* is a prerequisite to facilitate processes, and it influences the outcome required to achieve PCC. It is linked to the key roles of governments and organizations within and outside healthcare services. In this study, the component of structure is seen in the light of how maternity care is provided, particularly including the presence of strong, necessary, and practical family support during the new mother’s stay in the delivery ward. Other important structures are the education of professional midwives and the organization of HEWs. The *process* in PCC includes the development of communication between midwives and birthing women together with respectful and compassionate care. *Outcomes* are identified through the evaluation of access to care in the area, including timely care, knowledge of the availability of facility-based delivery care, and patient-reported care experiences.

A literature review of studies from developing countries reveals that interpersonal behavior is crucial for women’s satisfaction with maternal healthcare.^11^ According to previous research from Ethiopia and other developing countries, women often report satisfaction with the care they receive based on the knowledge that the service is beneficial and potentially life-saving.^12-14^ Additionally, women value the support and reassurance of professionals who are sensitive to their needs during childbirth.^15^ Their families tend to share these positive experiences, which leads others to give birth in healthcare facilities, as well.^16^ Nevertheless, there are barriers against the use of healthcare facilities with skilled birth attendants. Mothers may be concerned about the healthcare workers’ interaction skills,^14,5,17,18^ and structural challenges, such as limited resources and technical factors.^17^ The studied region is characterized by scarce material resources, including delivery wards and access to medicine.^19^ Despite such barriers, 47.6% of all birthing women in the region delivered their babies at a healthcare facility in the past 5 years.^2^ Comparatively, 95.5% of women in the same region had home births from 1995 to 2000.^17^ This increase seems to be related to the use of ANC services, mothers’ education, and household income.^19^

Although women are generally satisfied with their care, many studies have identified barriers such as disrespect, neglect, abuse (often, verbal abuse),^12,20-23^ and women’s limited decision-making power.^18,24-27^ Few studies have explored women’s experiences in the context of childbirth in primary healthcare facilities, and women’s descriptions of their experiences after using delivery ward services are even scarcer. This study aims to learn about women’s birthing experiences and their thoughts about important aspects of delivery care in professionally staffed healthcare facilities.

The research question in this study was, “How do women in a rural southern district of Ethiopia experience maternity care offered at the local delivery wards?” This is important to understand how women want maternity care to be designed to meet their needs better.

## Methods

A qualitative exploratory inductive design was chosen, with individual interviews and observations, to better understand the underlying reasons, opinions, and motivations for giving birth at a healthcare facility. In collaboration with Hawassa University, the study was conducted in Hawassa Zuriya Woreda (district) whose city, Dore Bafano, is located approximately 300 km south of the country’s capital, Addis Ababa. In 2018/2019, the population of this woreda was approximately 165 000. The woreda has 1 public primary hospital and 4 public healthcare centers. The maternal services are free. Only 1 ambulance is available in the entire district.

### Data collection

Interviews were conducted in February 2019. The interviewees were recruited by a research assistant who identified them from delivery books at healthcare facilities. Local HEWs helped locate women in various communities who fulfilled the inclusion criteria listed below. All the women interviewed had given birth in the past 8 months.

*Inclusion criteria*: The interviewees had to be birthed at least 1 child at a public healthcare facility in the district studied. They had to communicate orally and provide informed consent. *Exclusion criteria*: Potential interviewees with concomitant serious medical problems or those diagnosed with a psychiatric condition were excluded. Nineteen women were interviewed (Table 1) about their perinatal experiences, preferences during labor, and the postpartum period at the healthcare center or public primary hospital. During the interviews, an interpreter translated from English to Sidaamu Afoo and back to English and an interview guide with open-ended questions was used. After some demographic questions, the women were asked to share their perinatal experiences. Examples of the open-ended interview questions were: “Please tell me about your planning of the childbirth,” and “Describe how you experienced being taken care of during childbirth and the hours afterward.” We added questions based on the women’s responses. After 19 interviews, the data seemed saturated, as the last few interviews did not provide new insights, and no new nodes were derived from them.

**Table 1.** Demographics of women participating in individual interviews (n = 19).

Age	15-25	12
	26-35	4
	Unsure	3
Number of children	1-3	18
	4-6	1
Distance to a healthcare facility	Up to 30 min walk	12
	½-3 h walk	7
Total stay in a healthcare facility	Less than 24 h	13
	1-3 days	6
Educational level	Primary school, 1-8 grade	12
	Secondary school, 9-12 grade	5
	University/college	2

The interview guide was pilot-tested, demonstrating that no changes in the guide were required. Data from these pilot interviews were not included in this study. Following the interviewees’ wishes, all but 2 women were interviewed in their own homes. The latter 2 were interviewed at the local healthcare center.

The first author conducted all the interviews, and a trained female research assistant who spoke both English and the local language fluently served as a translator. The interviews were audio-recorded with the participant’s permission.

Observations of deliveries were also conducted to study the process in its natural setting, thereby providing a richer understanding of the mothers’ behavior and experience during their stay in the maternity ward. This method is essential for gaining insight into naturalistic settings and their members’ ways of viewing them. Two slightly different maternity wards, at a healthcare center and primary hospital, were chosen to obtain varied observation data.

All the observations of the birthing women were conducted by the first author, an experienced midwife. The observation period was January 2020. The observed birthing mothers had to agree to the observation, and a professional birth attendant was required to be present at the facility. Women were observed during and after delivery. The focus was on women’s behavior and their relationships and communication with healthcare providers and networks. Notes on what activities took place, the physical setting, social interactions, and individuals present were taken during and shortly after the observations. Since many childbirths occur at night, the observer stayed at the healthcare facility around the clock for 4 periods of 2 to 5 days each, totaling approximately 200 h. This field study offers in-depth insights into the functioning of wards.

### Data analysis

The interviews were electronically recorded, transcribed verbatim, and translated into English. We performed a quality check of the translations through an independent translator who spoke both English and the local language. We used thematic content analysis,^28^ and, thus, an inductive approach. (1) We read the transcribed interviews and observation field notes several times to acquire an overview of the data. (2) We reviewed the data, noted initial ideas, and coded interesting characteristics across the 2 datasets (interviews and observations). Three codes were collated into potential themes with the initial steps performed by the first and last authors, who sorted the codes into main and subthemes. The analyses were discussed by all authors. (3) A thorough review of the initial themes was conducted. (4) Two main and 5 sub-themes were defined and named. (5) The preliminary text was developed collaboratively through discussions by all authors. Although the analysis is described in steps, this was not a linear process and we returned to earlier steps severally to ensure that the results were grounded in the collected data. The NVivo version 12 software program was used for data coding. Transparency was ensured through a thorough description of the analytic process from “raw” data to themes. Rigor and trustworthiness were safeguarded through discussions between the first and last authors, in which they critically questioned their influence on the interpretation of the data and the formulation of themes. Analytic credibility was ensured through the choice of context, participants, and a research approach suitable for the focus of this study. Quotations are presented to emphasize our findings.^29^

### Ethical considerations

The women received a written invitation to participate in the study, and each participant signed an informed consent form. The establishment of mutual respect and trust between the researcher and interviewees was emphasized. Ethical approval was granted by Hawassa University (Ref. no. IRB/008/11), and the Norwegian Centre for Research Data (Ref. no. 58985). The managers of the healthcare facilities were informed about the project through a letter from the regional woreda (district) office. The study was conducted per the guidelines governing research ethics, such as informed consent, the principle of voluntariness, and estimating the risk against the benefit of the study29. The data material was treated confidentially, and anonymity was secured.

## Results

All 19 in-depth interviews and observation data were included in the analyzed material. This resulted in 2 main themes, each with several subthemes. The 2 main themes were as follows: (1) increased awareness and safety were the primary reasons for giving birth in a healthcare facility and (2) traditions and norms affected women’s birth experiences.

### Increased awareness and safety—reasons for giving birth in a healthcare facility

The majority of the women said they were happy with the maternity care they received and used *safe childbirth* as the key term to describe why they preferred skilled birth attendants. They believed that women who gave birth at public healthcare facilities were likely to survive without lasting injuries. To describe why, one of the women said: “They ordered me to walk around during labor, explaining that movement facilitates the labor process. Professionals gave me intravenous fluids because blood was lost after excessive vaginal bleeding. They helped me.” When asked what extra value they received from giving birth at a healthcare facility, most women pointed to the professional treatment, especially in case of birth-related complications.

#### Women ask for competent midwives to prevent risk

Professionals used their knowledge to uncover possible complications at an early stage. The interviewees reported that midwives worked to prevent risks or acted quickly if birth-related complications occurred. However, they did not prevent any possible risks. One of the interviewees’ first childbirth experiences at a healthcare center was stressful. Although the midwife had referred her to a hospital about 45 minutes away by ambulance, they could not save her newborn boy. Despite this, she said she felt safe when she gave birth to her second baby, as the midwife knew about her previous experience on arrival, which made her feel less vulnerable.

Most of the women expressed great respect for the care offered to ensure safe childbirth. They relayed how the midwives helped them during severe bleeding, prolonged labor, or when they had abdominal pain. Observations in the delivery ward confirmed this, although we did not observe any women receiving analgesics during the active labor phase. Only 1 woman was observed to be receiving a local anesthetic when the midwife sutured an episiotomy.

#### Most women feel well taken care of

Most women said they were treated with respect and received material, supportive, and communicative care during childbirth.

Their stories about the quality of the care they received were often quite detailed and tended to focus on respectful treatment and care that gave them a feeling of well-being:

“They cleaned my uterus well; they were good with me.”

“. . . they asked me about how I was feeling.”

“. . . and they even changed my soiled clothes.”

The experience of being somewhat in control was also helpful to women who found themselves in vulnerable situations: “They told me about the remaining time of delivery, how the labor progressed, and reassured me” one woman said. Most of the women received guidance during labor: “During my stay (at the healthcare center), the professionals told me what I should do and avoid regarding labor to prevent further problems. They missed their sleep for me.” Another woman explained that “they taught me how to breastfeed, and they showed me how to attach to the newborn.” All the interviewees seemed to trust the professionals’ competence during the various stages of childbirth.

#### Inadequate care and lack of respect

Although many expressed an overall positive experience with the care they received, some said that their expectations were not fully met. A central negative issue was the shortage of materials. For instance, one interviewee reported that “they did not give me any medicines; they did not follow me up the way I expected. I want the healthcare professionals to do more than they did to improve the health of mothers and children.” Other examples were that the ambulance did not arrive in time, they did not receive any care at night, or they generally felt neglected by the professionals: “A new midwife assisted me, but she did not give me adequate and satisfying care. She only prescribed analgesics for me and left the ward while I was there.” Some said that friendly and polite care was lacking, with 1 woman summarizing her experience this way: “The way they treated me made me angry.”

Observations indicated that midwives tended to focus more on medical treatment than on support and care. While the midwives took great care of the administration of medicine and during the active phase of childbirth, we did not often see damp cloths placed on the mothers’ foreheads or hear comforting words spoken to them when the pain was intense. Often, the professionals did not inform the women before commencing medical procedures. For example, they were observed to impatiently force women’s legs apart when conducting a vaginal examination. Communication during childbirth tended to be most from the professional to the woman, and rarely the other way around.

### Traditions and norms affected the women’s birth experiences

Safety and the quality of care were not always stated as the reasons for giving birth at a healthcare facility: “Nowadays, giving birth at the healthcare center is becoming part of the culture, because women are being taught about health and related issues,” an interviewee said. One of the women simply said that she followed the lead of other pregnant women. Several women explained that, in recent years, the women of their local community had changed their preferences about where they wished to give birth. Access to skilled birth attendants and knowledge of the benefits they provide gradually changed their preferences. Most of the women said they chose a public healthcare facility to deliver with their husbands. In some cases, the entire family is involved in the decision. Nonetheless, “The final decision was mine.”

#### Male professionals: A challenge for some birthing women

Although none of the women were observed asking for a female midwife when a male came to care for them, for some, being assisted by a male midwife was a challenging experience: “[When] the male caregiver came to assist me I hesitated because I am afraid of men.” Another interviewee said she shared her positive experience about receiving care and treatment from a male birth attendant to teach another woman that male midwives are professional healthcare workers as well. Someone told her that:

“Male professionals will examine our pelvis with their hands; sometimes, an episiotomy may be considered; we do not want to open our body to them. I tried to tell them the reality that, after death, anyone can see our body. It is better to show our body to others rather than dying ashamed to be exposed for examination purposes.”

One of the women said, “If the government has approved and allowed them to serve us, I am okay with male or female professionals.”

#### Trust in God

Trust that God was with them and would help them during childbirth, regardless of where they gave birth, was another reason cited by the women for their feelings of safety. Common sentiments were:

“God had provided the maternity care.”

“I have left it in the hands of God . . .”

“It was good; only God helped me during the labor process.”

The women added that their families prayed to God and thanked Him for their healthy childbirth outcomes.

## Discussion

This study aims to learn about women’s experiences and perspectives on important aspects of delivery care at healthcare facilities. These findings are discussed in light of the outcomes, structure, and process outlined in Santana’s PCC model,^5^ which we found suitable for the discussion.

The *structure* is described as the healthcare system and context, which constitutes the framework.^5^ Despite the model and structures characterized by scarce resources and somewhat unpredictable ambulance service, most of the mothers said they were well taken care of by the healthcare professionals. It is difficult to offer quality maternity care when necessary equipment is lacking and when the workload is too heavy for the available healthcare staff.^17^ Our findings strongly indicate that structured group meetings organized by HEWs or other professionals, as well as traditional coffee ceremonies in the local communities, are well-established meeting places for the sharing of information and experiences. The mothers reported that these ceremonies, both official and private, supported their decisions to give birth at a healthcare facility. An *outcome*, as identified in the conceptual framework of PCC, depends on having the necessary structures available for professionals to provide adequate care to birthing women.^9^ Access to timely maternity care is identified as a crucial factor for reducing maternal mortality together with knowledge about what professional care can offer.^15^ Even though theoretically, the healthcare facilities in the studied district should have delivery care available 24 h a day, 7 days a week, the midwives sometimes left the birthing women to fend for themselves because of staff shortages or poor human resource management. The midwives had to get to bed after long, tiring shifts, which could last up to 24 h. If adequate access to healthcare is to be ensured, 1 ambulance is far from adequate for this large a district. Additionally, the inability to afford necessary medicine makes adequate treatment inaccessible. Another important structural condition is the development of midwifery programs at Ethiopian universities, in which the enrolment of male students is currently common. However, the attitude of Ethiopian women toward male caregivers has been cited as a potential barrier for men being employed as professional birth attendants, since many women are neither used to nor comfortable with being treated and cared for by men other than their husbands.^20,23,30^ Therefore, our participants’ experience of being comfortable with male midwives was unexpected, demonstrating that this opinion among mothers is gradually changing.

The women reported that they trusted in God; some even explained that God had given them this facility-based delivery care service. Faith in God seemed to be a strengthening factor through a challenging childbirth process, regardless of the context in which maternity care was offered. The importance of God was also mentioned in a study that included women who gave birth at home.^30^ Furthermore, the importance of religious beliefs and trust in God is emphasized in a systematic review in which researchers identified that women in rural areas in Africa preferred healthcare facilities where their religious beliefs were respected and religiously sensitive care was provided.^31^ Understanding the patient’s cultural context and the importance of religious and spiritual factors are important *process* elements in PCC^5^ including the level of healthcare, which can be improved by providing a supportive environment, respectful care, and an open and trusting dialog between women and midwives, even when resources are scarce. Safe, respectful, and compassionate care is important. Previous studies have indicated that midwifery care in low-income countries is primarily experienced as safe.^12-14^ These findings are in line with our data. A birthing mother’s feeling of safety probably reflects the fact that midwives are trained to save lives. It is noteworthy that a mother’s feeling of safety is also closely related to her experiencing some control during the childbirth process, depending on whether continuous information is provided. This made the women partners in making informed decisions while on the delivery ward, a result also found in a quantitative study from Ethiopia.^29^ When caregivers provided updates on the status and progress of labor and explanations about what was being done, this was significantly associated with satisfaction with the care provided. Bradley et al^20^ concluded in a qualitative, systematic review that psychosocial elements are essential to a woman’s birth experience. While a study by Bulto et al.^30^ indicated that midwives cultivate positive communication with women, another Ethiopian study revealed that healthcare providers tend not to ask female patients for their consent before performing medical procedures.^31^ The quality of the communication is important for women to feel respected. This is in line with the International Code of Ethics for Midwives,^32^ which states that every woman, regardless of their background, must be met and treated with respect. However, several studies indicate that women’s feelings are being hurt, and they must endure inappropriate comments and lack consistent care while giving birth. Studies from Ethiopia and other countries indicate that birthing women may experience disrespectful care.^12,21,23,33^ We wonder whether women’s limited expectations regarding their level of care in the delivery ward might be a reason why most of our interviewees were satisfied. This is despite having made several observations of poor and 1-sided communication on the part of midwives. The PCC framework is not limited to the patient but includes both caregivers and families.^9^ While our observations confirmed that the midwives had the sole responsibility for the care of women during the actual expulsion in the second stage of labor, the rest of the care was entrusted to the family. In collectivistic cultures, as in Ethiopia, the woman’s extended family and network are expected to be strongly involved in essential processes such as childbirth. The collectivistic way of life was thus evidenced in the rural healthcare facility and the delivery ward, as in the women’s life in general.

### Strengths and limitations

Nineteen interviews of birthing women, together with observations of births over several periods, are some of the strengths of this study, although the research was conducted in a limited geographical area in a large and highly diverse country. An example of a limitation is that the first author conducted this study in a culture that was quite different from her own. However, she is a trained midwife who is aware of her cultural preunderstanding. She has visited the district studied several times over the past 8 years and thus, has gained valuable insight into the local culture. Nevertheless, without the input and guidance from the second author, who is a lecturer at Hawassa University, and the local midwives, this study would have been inadequate to fully appreciate the cultural context. The interviews were conducted in the local language with the help of a fluent bilingual translator. Since meaning may be lost in translation, linguistic problems were discussed as they occurred during the interviews and later during the translation process.

## Conclusion

The interviewed women seemed to be mostly satisfied with their care, but several had experienced insufficient and disrespectful care. Our findings indicate that traditions and norms influenced women’s experiences of facility-based childbirth. Both the healthcare systems, managers, and midwives should ensure that the birthing women receive high-quality, culturally adapted, timely, and respectful care to meet their needs. Even expectant mothers cite safety as the main reason for their choice of professional care, these women not only need to survive childbirth but also remain healthy and thrive. If childbirth assisted by skilled birth attendants is a goal for the future, challenges regarding inadequate human resources and supplies must be solved. Further research is required on the development of maternity care in rural areas that involves the safety of both mother and child, and the psychological and spiritual aspects of childbirth.
